# 

**Published:** 1864-12

**Authors:** 


### CONTENTS.

### ORIGINAL CONTRIBUTIONS.

ART. XLI. Bromine in the Treatment of Diarrhœa and Dysentery. By H. F. Gilbert, Executive Officer, ........ 657
ART. XLII. Opium and Quinia Sulpatis. Their Antagonism and Co-operation. By T. D. Fitch, M.D., of Chicago, ........ 661
ART. XLIII. Rheumatic Irritation in the Diaphragm and Psoas Muscles.—Clinical Cases, Reported to the Chicago Medical Society, Dec. 23, 1864. By N. S. Davis, M.D., of Chicago, Ill., ........ 665
Correspondence from New York, ........ 670

### SELECTIONS.

Report of Cases of Hospital Gangrene. By William Thompson, M.D., Assistant-Surgeon, U.S.A., ........ 674
On the Physiological Effects of Tobacco. By Dr. Richardson, ........ 697
The Treatment of Albuminuria in Children, ........ 701
The Degeneration and Regeneration of the Nerves, ........ 704

### EDITORIAL.

Chicago Medical Society, ........ 706
Mortality of Chicago, ........ 709
Profits of Medical Practice in Chicago, ........ 711
Transactions of the Illinois State Medical Society, ........ 712
Illinois State Medical Society, ........ 713
The Yellow Fever in Newbern, N. C., ........ 713
Poisoning by Tobacco Leaves.—By Dr. Namias, ........ 714
The Arteria Innominata Successfully Tied, ........ 714
Sudden Death in Contusions and Fractures from Pulmonary Emboli, ........ 715
Fibrinous Coagula of the Heart, ........ 715
Surgeon-General of New York, ........ 716
Ligating the Arteria Innominata, ........ 716
Oxygen Gas, ........ 716
Proportion of Births to Population, ........ 716

